# The Effects of the Pilates Method on Pelvic Floor Injuries during Pregnancy and Childbirth: A Quasi-Experimental Study

**DOI:** 10.3390/ijerph18136995

**Published:** 2021-06-30

**Authors:** Carmen Feria-Ramírez, Juan D. Gonzalez-Sanz, Rafael Molina-Luque, Guillermo Molina-Recio

**Affiliations:** 1 Departamento de Enfermería, Universidad de Huelva, 21071 Huelva, Spain; carmen.feria@denf.uhu.es; 2 Centro de Investigación en Pensamiento Contemporáneo e Innovación para el Desarrollo Social (COIDESO), Universidad de Huelva, 21071 Huelva, Spain; 3 Grupo Asociado de Investigación Estilos de Vida, Innovación y Salud, Instituto Maimónides de Investigación Biomédica de Córdoba (IMIBIC), 14004 Córdoba, Spain; Rafael.moluq@gmail.com (R.M.-L.); en1moreg@uco.es (G.M.-R.); 4 Departamento de Enfermería, Farmacología y Fisioterapia, Facultad de Medicina y Enfermería, Universidad de Córdoba, 14041 Córdoba, Spain

**Keywords:** nursing, midwifery, nurse, Pilates method, pelvic floor, injuries, episiotomy

## Abstract

The perineal injuries suffered during childbirth have a great impact on the quality of life of the female population. Evidence suggests that the Pilates method is used by pregnant women to improve the physical and psychological outcomes of pregnancy. The aim of this study was to investigate the influence of the Pilates Method during pregnancy on the incidence and degree of intrapartum perineal trauma. A quasi-experimental study was carried out between November 2018 and December 2019 at different health centers in two health districts. Participants were 72 pregnant women attending the antenatal program, who were assigned to a Pilates group or a control group (48 and 24 pregnant women, respectively). The main outcome measurement was perineal trauma during childbirth. After participating in the Pilates program, the women in the experimental group were significantly less likely to suffer perineal trauma in spontaneous deliveries compared to the women in the control group. After evaluating these results, it is concluded that health center managers should promote the training of midwives in the prevention and treatment of pelvic floor injuries during pregnancy and should consider strategies to enhance adhesion and participation with respect to pelvic floor exercise programs throughout pregnancy by means of Apps and other digital media specifically aimed at this phase.

## 1. Introduction

It is well known that female pelvic floor (PF) weakness and/or pelvic floor dysfunction (PFD) can have both structural and functional effects, such as urinary incontinence, bowel incontinence, pelvic organ prolapse, and dyspareunia and/or sexual dysfunction [1,2,3,4]. One of the main risk factors associated with pelvic dysfunction is the reproductive process, including pregnancy and birth. During this process, both modifiable and nonmodifiable risk factors for PFD can be identified. Chief among the former are the pregestational and full-term body mass index (BMI), weight gain, smoking, the type of birth, the use of forceps, the duration of the first and second stages of birth, the practice of episiotomy, and the use of epidural anesthesia. Among the nonmodifiable risk factors (also known as risk indicators) are age at maternity, position of the fetus and circumference of the newborn’s head, the weight of the newborn, and the presence/existence of perineal injury, chiefly those which can affect the anal sphincter [1,3].

Of all these factors, vaginal birth is the chief modifiable risk factor for developing PFD, as women in this group are 2.8 times more likely to suffer stress urinary incontinence, and 5.5 times more likely to suffer a prolapse of the pelvic organs, in comparison to those who give birth via caesarean section. These risks increase with instrumental delivery, which seems to be related to the fact that the incidence of injury to the anal musculature is higher in this procedure [5].

Among the nonmodifiable obstetric risk factors, chief is perineal trauma, whether spontaneous or induced (second-degree tears or obstetric anal sphincter injuries), which represents one of the most frequent complications associated with birth (85% of puerperal women) and has a clear influence in the subsequent appearance of PFD [6,7].

Although in recent years the practice of routine episiotomies has decreased in Spain, the percentage remains above the recommendations of the WHO at around 40.2% [6,8,9].

Perineal wounds suffered during birth significantly increase the risk of PFD, with a consequent impact on the quality of life of female population [1,2,3,10].

Primary prevention of PFD is therefore essential, and the following interventions have been shown to be effective: (1) perineal massage during pregnancy [11], (2) weight management and the promotion of physical exercise [12,13], and (3) PF strengthening exercises, which help to reduce discomfort and reduce the risk of urinary incontinence in the third trimester of the pregnancy and after childbirth [3,6].

With respect to exercises for strengthening the PF, the most common are hypopressive abdominal exercises and the Pilates Method (PM), which focus on developing the musculature of the transverse abdominis and pelvis in order to decrease intra-abdominal pressure [13]. 

Pilates is based on control, strength, and flexibility. It focuses particularly on the abdominal muscles, vertebral column, and PF, and is thus instrumental in improving body alignment and good posture [14,15,16,17]. Advocates also note that practitioners gain a greater personal health, among other benefits [18,19]. 

Given the marked increase in practicing Pilates during pregnancy, various studies have been carried out to evaluate its effectiveness [14,16,20]. With regard to the specific relation of Pilates with PFD, two studies found a positive impact on the PF musculature during pregnancy [21,22], while another study concluded that the method was a valid means of helping to prevent the dysfunction [23].

The starting hypothesis of this study was that the incidence of perineal wounds during childbirth would be lower among those participating in a specially designed Pilates program. Therefore, the aim of this study was to evaluate the influence of Pilates sessions during pregnancy on the incidence and degree of intrapartum perineal injuries.

## 2. Materials and Methods

### 2.1. Study Design

This study was a 4-week multicenter quasi-experimental trial conducted from November 2018 through December 2019. Pregnant women who received routine antenatal care in health centers (HCs) pertaining to two distinct health districts were eligible to enroll.

### 2.2. Sample/Participants

All pregnant women who were attending antenatal classes at HCs in two distinct districts were informed by the midwife of the possibility of taking part in the study if they met the inclusion criteria. The inclusion criteria were as follows: (1) being registered on an antenatal program (AP), (2) giving written consent of participation, (3) the pregnancy being a singleton, (4) the pregnancy being low risk [24], (5) there not being any contraindications for physical exercise, and (6) being at least 18 years old.

Women who had missed antenatal appointments, had difficulty in speaking or understanding Spanish, had given birth by caesarean section, or declined to participate were excluded from the study.

#### 2.2.1. Interventions

The study was carried out in two stages: the first involved finding Pilates trainers to deliver the sessions, while the second collated participant data from both the experimental and control groups. The first phase took place in November 2018, with the collaboration of two trainers in Huelva and one in Seville, all with the same training background. The second phase was carried out between December 2018 and December 2019 in the corresponding HCs in Huelva and Seville.

In order to eliminate potential bias as a result of the antenatal classes being delivered by health professionals of different categories, only those HCs where classes were delivered by a midwife were selected for the study. The women participating in the corresponding antenatal classes at the respective centers were then invited to participate in the study, and assigned either to the experimental group (AC + PM) or to the control group (AC only).

#### 2.2.2. Experimental Group

The women in the experimental group received two one-hour Pilates sessions per week over a period of 4 weeks (Appendix A. In addition, the participants received their usual antenatal classes at their respective HCs in accordance with the Comprehensive Healthcare Program for Pregnancy, Childbirth, and Postpartum (CHPPCP) from the Andalusian Regional Government [24].

#### 2.2.3. Control Group

The women in the control group received solely the antenatal classes at their corresponding centers as programmed (Table 1).

#### 2.2.4. Sample Size

A minimum sample size of 36 women was originally projected, for a confidence level of 95% and a power of 80%, considering 49% avoiding episiotomy in the control group in comparison with 98% in the experimental group [25], and divided according to a ratio of 2 women in the control group for every woman in the experimental group. This number was increased in case any women dropped out, such that the final number of participants was 72, of which 24 pregnant women formed the experimental group and 48 pregnant women the control.

### 2.3. Data Collection

#### 2.3.1. Outcomes

The outcomes variables were age (years), blood pressure (mmHg), weight (kg), BMI (kg/m^2^), starting level of physical activity, and tobacco use. These variables were all measured by experienced personnel at the start of the experimental phase, at two weeks, and again at four weeks after the treatment for both groups had been completed.

Variables relating to childbirth, birthweight (kg), and weight gain during gestation were measured between the eighth and tenth day after birth by telephone interview and review of hospital medical history. To calculate the weight gain (kg), the weight measured in the first trimester and the weight prior to delivery were taken as reference. The numbers of weeks of pregnancy at birth were measured on a discrete quantitative scale. Labor onset (spontaneous, stimulated, or induced), type of delivery (spontaneous, assisted delivery with forceps, Thierry’s spatulas or vacuum extraction, or caesarean section), the use of intrapartum pharmacological analgesia (none, epidural anesthesia, sedatives, or nitrous oxide), and type of episiotomy (not required, median, lateral, or medio-lateral) were measured by nominal scales, while the degree of perineal tear was evaluated on an ordinal scale (no injury; first degree: laceration of the vaginal epithelium or perineal skin only; second degree: involvement of the perineal muscles but not the anal sphincter; third degree: disruption of the anal sphincter muscles, which is further subdivided into grade 3a: less than 50% thickness of external anal sphincter torn, grade 3b: more than 50% thickness of external anal sphincter torn, and grade 3c: internal anal sphincter also torn; fourth degree: a third-degree tear with disruption of the anal epithelium). 

Weight (kg) and height (cm) were recorded during the routine antenatal appointments at the HCs using stadiometers with weight scale function. The level of physical activity was measured using the International Physical Activity Questionnaire (IPAQ) [26].

#### 2.3.2. Validity and reliability/Rigor

The TREND recommendations were followed in the design and development of this research. To avoid bias in the assessment of the results, the professionals who assessed the progress of labor in the delivery room and completed the medical history did not know whether the woman belonged to the intervention or the control group.

#### 2.3.3. Ethical Considerations

The study was carried out in keeping with the principles enshrined in the Declaration of Helsinki (1964), the Convention for the Protection of Human Rights and Dignity of the Human Being with regard to the Application of Biology and Medicine (1997), and the Universal Declaration on the Human Genome and Human Rights (1997), and also complied with the requirements stipulated by Spanish Law 3/2018 of the 5 December in the area of biomedical research, data protection, and bioethics. Approval from the bioethics committee of the Andalusia Health Service (SAS) was also obtained. Only data for which informed consent had been given in writing were used.

#### 2.3.4. Data Analysis

The quantitative variables are presented with the mean and standard deviation, while the qualitative data are given in frequencies and percentages.

In order to measure the goodness of fit of the quantitative data to a normal distribution, a Lilliefors-corrected Kolmogorov–Smirnov test was used. Bivariate analysis was carried out with Student’s *t*-test for the comparison of two means. In the case of the qualitative data, the chi-square test was used, except when Fisher’s exact test was required. Likewise, for the analysis of three or more means, a repeated-measures ANOVA was used to evaluate the effects of the treatment in the two groups, at baseline and at three and five weeks. Correlation between the quantitative variables was verified using the Pearson coefficient correlation (*r*). Finally, in order to allow for the possibility that the data did not meet the criterion of normality or homoscedasticity, nonparametric versions of the tests above were also carried out.

Binary logistic regression models adjusted for various qualitative and quantitative predictive variables were calculated in order to determine association of the variables with perineal trauma in childbirth.

The odds ratios (ORs) were determined with a confidence interval of 95%. The goodness-of-fit tests (–2 log likelihood, goodness-of-fit statistic, Cox and Snell *R*^2^, Nagelkerke *R*^2^, and Hosmer–Lemeshow) were calculated to evaluate the overall fit of the model.

In all statistical analyses, alpha was set to below 5% or the significance level was established at 5% (*p* < 0.05) and a confidence interval of 95% was computed. All statistical calculations were made using IBM SPSS Statistics version 25.0.

## 3. Results

**Figure 1:**
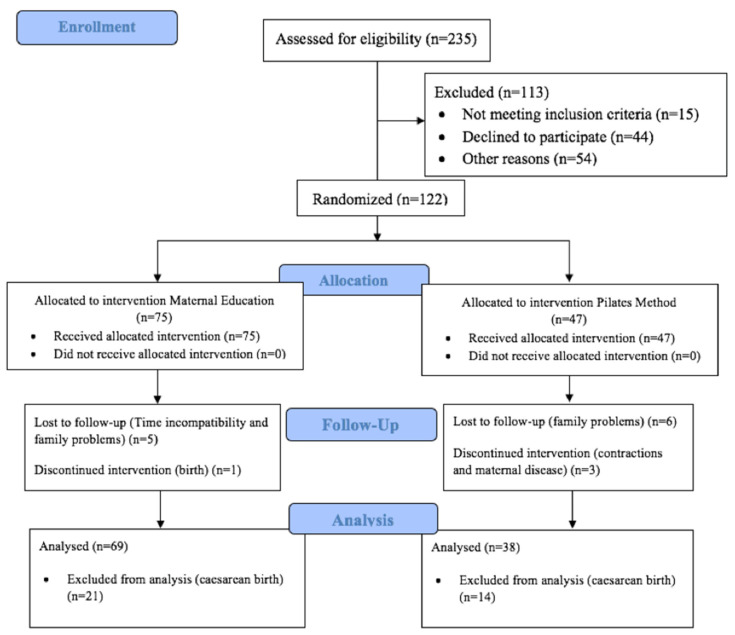
The flow diagram of the participants through each stage of the study.

Over the period in which the study took place, 294 women attended the AC sessions in the participating health centers. After inviting those who met the selection criteria to participate, 47 women were chosen to form part of the experimental group and 75 women the control. Nine women from the experimental group were forced to drop out for reasons of family problems, contractions, or maternal illness, while six dropped out from the control group because of family problems, clashes with the schedule, and giving birth. It should also be noted that all participants who ultimately had a caesarean section (21 women in the control group and 14 women in the experimental) were excluded from the data. The final numbers of participants were 24 in the experimental group and 48 in the control. A flow chart of the participants at each stage of the study can be seen in Figure 1.

### 3.1. Characteristics of the Sample 

Of the 72 women who finally participated in the study, 24 attended the PM sessions (experimental group), while 48 attended solely the usual AC (control group). The average age of participants at the start of the study was 32.4 (5.2) years old; body mass index was 25.2 (3.8), indicative of slight overweight; systolic and diastolic blood pressure values were 110.8 (10.5) and 67.6 (8.5), respectively, and lifestyle habits involved low physical activity (45.8%) and generally no tobacco use (94.4%). With respect to the gestational age at the point of recruitment to the study, the average of all participants was 27.3 (3.7) weeks. For none of these variables were significant differences found between the women in the experimental group and those in the control group. More detailed information on the baseline condition of both groups can be found in Table 2.

### 3.2. Onset of Birth, Prevalence of Perineal Trauma and Predictive Variables

In terms of delivery, it can be noted that most were classified as dystocial (70.8%), a circumstance that had no association with the presence or absence of perineal laceration. No differences were found with respect to the use of analgesics (administered in 83.3% of the cases), the gestation week in which the birth occurred (39.2 (1.4) weeks among those suffering this complication, as opposed to 38.9 (1.5) among those who did not), or whether the birth was induced (19.4%) or spontaneous (80.5%). Table 3 shows the data concerning the onset and progress of labor with respect to the appearance/non-appearance of laceration, and Table 4 shows the data concerning the weight gain.

Of the 72 participants, tearing of the vaginal or surrounding tissues occurred in 30 cases, a frequency of 41.7%. The results indicate that this proportion was lower among those women who underwent an episiotomy, with laceration occurring in 9.7% of the cases, as opposed to 65.9% in the case of those who did not undergo the procedure (OR, 0.06; 95% CI, 0.01–0.21). Likewise, participation in the Pilates sessions was demonstrated to be effective, with this complication occurring in the majority of cases (86.7%) among the women receiving solely the usual antenatal classes (OR, 0.17; 95% CI, 0.05–0.57) (Table 3) and laceration occurring with a frequency of 54.2% in this group, in contrast to 16.7% among those who received the Pilates sessions (*p* = 0.006).

The other variables, namely, age, weight, BMI, blood pressure, level of physical activity, tobacco, weight gain during gestation, and birthweight, showed no association with the occurrence of laceration during labor (Table 3).

Furthermore, when the association of the different variables with this complication was studied using a multivariate binary logistic regression model, it was found that both the performance of an episiotomy and participation in the Pilates sessions have a protective effect against the occurrence of tearing. In addition, the variable education level included in the model indicated that not studied beyond primary or secondary education was associated with a lower probability of injury occurring (Table 5).

## 4. Discussion

This paper aims to evaluate the effectiveness of an intervention model based on the incorporation of an antenatal program of Pilates exercises as a means of preventing perineal trauma during childbirth and so reducing the incidence of female PFD.

The first thing to note is the high frequency of perineal trauma found in our sample (41.7%), of particular significance as these figures are used as an indicator of the quality of the healthcare system and the service it provides. Our results contrast with those of a systematic review, which estimates figures of 24% for second-degree tears and around 1.4% for third- and fourth-degree tears, both of which are more frequent in primiparae deliveries [27,28]. Nevertheless, as pointed out, many of the differences in findings between studies are directly linked to differences in healthcare practices at childbirth [29].

An example of this are the findings of D’Souza, in which perineal trauma occurred in more than 85% of vaginal births, consistent with the figure of 80% reported by Jansson, which also found first- and second-degree tears more frequent among primiparae, while third- and fourth-degree tears were more frequent among multiparae, at 3.2% and 4.3%, respectively [28,30].

As can be seen, there is a high degree of disparity among the data, and consequently the results cannot be extrapolated to other populations without taking into account the rate of episiotomies carried out in the health center or the health system in question. In short, the particular healthcare practices put into effect at childbirth are highly significant [31].

In the case of our study, the results show a lower incidence of laceration during childbirth (13.3% of the total) among those women who attended the Pilates sessions than those who attended solely the usual antenatal classes (86.7%), with a prevalence of 16.7% and 54.2%, respectively. These results support studies of interventions aimed at reducing perineal trauma through PF training programs, such as those described by León-Larios and Dieb. The former reports a figure of 17.6% for trauma in women who did not follow the program, compared with 6.9% in the case of those who did. In the latter study, 13.5% of the pregnant women who followed the PF training suffered trauma, as opposed to 21.5% of those who did not [6,32].

As can be seen, the results of these studies differ with ours, which may be due in part to the difference in the starting point of the intervention, from weeks 26 to 32, causing a difference in the women’s physical condition in the second and third terms. The duration of the training program might also be influential (between 4 and 8 weeks) since physical activity during pregnancy which promotes continuous strengthening of the PF muscles increases the probability of the perineum remaining intact during childbirth [31]. Another aspect that could account for the variability among the results is the combined use of other techniques for training/strengthening the PF, such as the incorporation of perineal massage [32].

Whatever the case, there is evidence to suggest that Pilates could be a tool for consideration as part of physical preparation programs for childbirth as it focuses muscular work on the abdomen and PF [22,33].

Another finding worthy of note is the association found between a higher level of education and an increase in the risk of trauma. This could be related to an increase in age at childbirth, although the finding needs to be researched in more detail in future studies as no previous study has been found which relates these factors.

On the other hand, the results concur with various previous studies in finding the practice of episiotomy an effective means of preventing tears during childbirth [29,34]. Nevertheless, this issue is controversial, as clinical practice differs greatly from one country to another, ranging from being a systematic practice in all births, as is the case in Argentina and Taiwan, mainly with first-time mothers [35], to being selectively performed in countries such as Sweden and Spain, where it is reserved for those cases in which the benefits outweigh the risks (imminent severe perineal tearing, prolonged second stage of labor, shoulder dystocia, instrumented delivery, and/or non-reassuring fetal heart rate) [28,29]. Episiotomies are still commonly practiced in instrumented births, despite there being little research supporting the benefits, as is also the case with spontaneous delivery with respect to a decrease in perineal pain, dyspareunia, urinary incontinence, or prolapse [29].

### Limitations

There are several limitations to this study that oblige us to treat the results with caution. Firstly, the study has a quasi-experimental design, and as such admits the possibility that the participants’ willingness to follow the Pilates program could be indicative of a greater concern for the health of the mother and unborn baby during pregnancy and so constitute a selection bias. Whatever the case, it is a type of bias recognized by the literature concerning this kind of experimental design [28]. Further, given that the multivariate analysis was able to minimize the possibility of confounding factors, we consider that the study could serve as a model for future experimental-based research. It would be of particular interest to take a larger sample, initiate data collection earlier, and include additional variables that might be associated with the occurrence of perineal trauma (obstetric history, number of previous deliveries, perineal massage prior to delivery, size of the newborn, duration of labor, etc.). Research into the effectiveness and outcomes of the practice of episiotomy, from both the clinical and maternal points of view, would also be desirable.

Finally, another under-researched area is the use of Apps and other digital media to encourage participants to see programs through to the end of their pregnancy. One of the few studies in this area reviews various PF training Apps (in the context of treating urinary incontinence) with a view to improving their adherence strategies [35].

## 5. Conclusions

In summary, although further research is needed into the sustained use of Pilates to develop the PF from the initial stages of pregnancy to childbirth, and its effects in preventing pelvic dysfunctions related to the pregnancy and giving birth, health center managers should promote the training of midwives in the prevention and treatment of pelvic floor injuries during pregnancy, and midwives should consider strategies to enhance adhesion and participation with respect to pelvic floor exercise programs throughout pregnancy by means of Apps and other digital media specifically aimed at this phase.

## Figures and Tables

**Table 1:** Schedule of sessions of the Maternal Education Program of the health service received by pregnant women participants.

Class Number	Theme of the Session
Session 1 (Initial measurement)	Physiological changes during pregnancy
Session 2	Childbirth
Session 3 (Intermediate measurement)	Care of the newborn
Session 4	The postpartum period
Session 5 (Final measurement)	Breastfeeding

**Table 2:** Demographic characteristics of 72 pregnant women who received prenatal care ^1^.

Variable	Total (*n* = 72)	Assistance to Regular Maternal Education (*n* = 48)	Assistance to Pilates Sessions (*n* = 24)	*p*-Value
Age, mean (SD)	32.4 (5.2)	32.4 (5.4)	32.5 (4.8)	0.80
Height, mean (SD), m	1.6 (0.0)	1.6 (0.1)	1.6 (0.1)	0.40
Weight ^2^, mean (SD), Kg	67 (10.1)	67.6 (10)	65.7 (10.3)	0.51
BMI ^3^, mean (SD), Kg/m^2^	25.2 (3.8)	25.2 (3.8)	25.0 (4)	0.45
SBP, mean (SD), mmHg	110.8 (10.5)	110.5 (11)	111.5 (10)	0.40
DBP, mean (SD), mmHg	67.6 (8.5)	67.6 (8.6)	67.6 (8.6)	0.28
Weeks of Gestation, mean (SD)	27.3 (3.7)	27.3 (4.1)	27.3 (2.6)	0.69
Physical Activity, *n* (%)				
Intense	33 (11)	4 (8.3)	4 (16.7)	0.42
Moderate	31 (43.1)	23 (47.9)	8 (33.3)	
Low	8 (45.8)	21 (43.8)	12 (50)	
Smoking, *n* (%)				
Nonsmoker	68 (94.4)	45 (93.8)	23 (95.8)	0.72
Smoker	4 (5.6)	3 (6.2)	1 (4.2)	
Educational level, *n* (%)				
Primary–Secondary.	26 (36.1)	18 (37.5)	8 (33.3)	0.59
Superior–Further.	46 (63.9)	30 (62.5)	16 (66.7)	

^1^ Abbreviations: BMI, body mass index; DBP, diastolic blood pressure; SBP, systolic blood pressure. ^2,3^ Weight and BMI before intervention.

**Table 3:** Characteristics of the sample of pregnant women participants by presence or absence of tearing and crude logistic regression ^1^.

Variable	Presence of Tearing (*n* = 30)	Absence of Tearing (*n* = 42)	OR Crude	IC 95%	*p*-Value
Age, mean (SD)	33.4 (5.2)	31.7 (5.1)	1.07	0.97–1.18	0.58
Weight, mean (SD), Kg	72 (11.7)	69.1 (10.3)	1.02	0.98–1.07	0.58
BMI, mean (SD), Kg/m^2^	26.8 (4.4)	26.1 (3.7)	1.04	0.93–1.17	0.53
Weight gain during gestation, mean (SD)	7.3 (6.7)	7,05 (8,0)	1.01	0.94–1.07	0.88
SBP, mean (SD), mmHg	111.9 (11.7)	110.7 (10.4)	1.01	0.97–1.05	0.50
DBP, mean (SD), mmHg	68.4 (7)	67.6 (7)	1.02	0.95–1.09	0.71
Physical Activity, *n* (%)					
Low–Moderate	27 (57.8)	37 (42.2)	1.22	0.27–5.53	0.59
Intense	3 (37.5)	5 (62.5)	1	1	
Educational level, *n* (%)					
Primary–Secondary.	9 (30)	17 (40.5)	0.63	0.22–1.7	0.17
Superior–Further.	21 (70)	25 (59.5.6)	1	1	
Weeks of gestation at the end intervention, mean (SD)	31.8 (4.7)	32.6 (4)	0.96	0.86–1.07	0.38
Smoker, *n* (%)					
Nonsmoker	2 (6.7)	2 (4.8)	1.43	0.2–10.75	0.89
Smoker	28 (93.3)	40 (95.2)	1	1	
Assistance to Pilates, *n* (%)					
Yes	4 (13.3)	20 (47.6)	0.17	0.05–0.57	0.007
No	26 (86.7)	22 (52.4)	1	1	
Weeks of gestation at labor, mean (SD)	39.2 (1.4)	38.9 (1.5)	1.18	0.82–1.6	0.70
Type of childbirth, *n* (%)					
Eutocic	5 (16.7)	16 (38.1)	0.32	0.10–1.02	0.19
Dystocic	25 (83.3)	26 (61.9)	1	1	
Type of labor, *n* (%)					
Induced	5 (16.7)	9 (21.4)	0.73	0.22–2.46	0.12
Spontaneous	25 (83.3)	33 (78.6)	1	1	
Labor analgesia, *n* (%)					
Yes	23 (76.7)	37 (88.1)	0.44	0.13–1.57	0.32
No	7 (23.3)	5 (11.9)	1	1	
Episiotomy, *n* (%)					
Yes	3 (10)	28 (66.7)	0.06	0.01–0.21	0.006
No	27 (90)	14 (33.3)	1	1	
Weight of newborns, mean (SD), g	3302.3 (347.5)	3135.6 (373.1)	1.00	1.000–1.003	0.006
*Pregnant women present at the end of Pilates (n = 24)/Pregnant women present at the end of education program (n = 48)* All deliveries were cephalic, and there is no record of posterior cephalic position in any of the records.					

^1^ Crude logistic regression. Abbreviations: BMI, body mass index; DBP, diastolic blood pressure; IC, confidence interval; OR, odds ratio; SBP, systolic blood pressure.

**Table 4:** Weight gain during gestation.

Variable	Total (*n* = 72)	Assistance to Regular Maternal Education (*n* = 48)	Assistance to Pilates Sessions (*n* = 24)	*p*-Value
Weight				
First trimester ^1^	63.3 (10.1)	63.7 (10.4)	62.5 (9.7)	0.62
Before the labor	70.4 (10.8)	70.6 (10.8)	70.2 (11.0)	0.88
BMI				
First trimester ^1^	23.8 (3.7)	23.8 (3.7)	23.8 (3.7)	0.97
Before the labor	26.4 (4.0)	26.3 (4.0)	26.5 (4.1)	0.89
Weight gain during gestation	7.2 (7.4)	6.9 (8.2)	7.7 (5.8)	0.63

^1^ First trimester: <10 gestational weeks.

**Table 5:** Binary logistic regression adjusted for age, level of education, episiotomy realization and assistance to Pilates sessions and presence of tearing as outcome variable ^1^.

Variable	Coefficient	OR	IC 95%	*p*-Value
Educational Level				
Primary–Secondary	−1.41	0.24	0.06–0.98	0.047
Superior–Further		1	1	
Assistance to Pilates				
Yes	−1.76	0.17	0.04–0.78	0.022
No		1	1	
Episiotomy				
Yes	−3.2	0.04	0.09–0.2	0.001
No		1	1	
Log-2 likelihood: 62.94; R^2^ Cox–Snell: 0.38; R^2^ Nagelkerke: 0.52; Hosmer–Lemeshow: 0.81, 5 gl (*p* = 0.98).				

^1^ Abbreviations: IC, confidence interval; OR, odds ratio.

## Appendix A

**Table A1:** Exercise Schedule of Pilates Method Program.

**Session 1**	**Session 2**	**Session 3**	**Session 4**
Squats Pelvis tilt Quadrupled (arms only) The Cat The archer The mermaid Side leg lifts Clam Side kick Leg circle	Squats Pelvis tilt Quadrupled (legs only) Arm rotations The cat The archer The mermaid Side leg lifts Clam Side kick Leg circle	Squats Side leg lifts Quadrupled (arms and legs) Clam Side kick Leg circle Leg lifts Pelvic curl Roll-up/roll-down Tiger The mermaid	Squats Quadrupled (arms and legs) Push up on knees The Cat The archer The mermaid Side leg lifts botton leg Clam Side kick Pelvis tilt Leg lifts Roll-up/roll-down Leg circle Back support
**Session 5**	**Session 6**	**Session 7**	**Session 8**
Squats Quadrupled (arms and legs) Push up on knees The cat Saw The mermaid Side leg lifts botton leg Clam Side kick Leg lifts Roll-up/roll-down Leg circle Tiger Back support	Squats Working arms with tape Quadrupled (arms and legs) Push up on knees The cat Saw Pelvis tilt The mermaid Side two legs Clam Side kick Leg lifts Pelvic curl Roll-up/roll-down Leg circle Back support	Squats Working arms with tape Quadrupled (arms and legs) Push up on knees The Cat The archer Saw The mermaid Side two legs Clam Side kick Leg lifts Pelvic curl Roll-up/roll-down Back support Leg circle Tiger Hamstream extension	Squats Working arms with tape Quadrupled (arms and legs) Push up on knees The Cat The archer Saw The mermaid Side two legs Clam Side kick Leg lifts Pelvic curl Roll-up/roll-down Leg circle Hamstream extension Tiger Side kick kneeling Back support
